# Id reaction and allergic contact dermatitis post-picosecond laser tattoo removal: A case report

**DOI:** 10.1177/2050313X211057934

**Published:** 2021-11-26

**Authors:** Ian TY Wong, Larry WK Cheung

**Affiliations:** The Skin Care Centre, Department of Dermatology & Skin Science, The University of British Columbia, Vancouver, BC, Canada

**Keywords:** Picosecond laser, autoeczematization, red tattoo ink, interface dermatitis reaction, laser tattoo removal

## Abstract

The novel picosecond lasers have emerged as a mainstay device in laser tattoo removal alongside Q-switch lasers, considered the gold standard in the field. Here, we present a 45-year-old female who developed a severe reaction to both her treated and untreated tattoos after two picosecond laser treatments and subsequent widespread eczematous eruption. Skin biopsies revealed findings consistent with hypersensitivity to exogenous red pigment. The clinicopathologic findings were consistent with an id reaction (autoeczematization) associated with allergic contact dermatitis to tattoo pigment. This case report highlights the potential for tattoo hypersensitivity following picosecond laser treatment and the dilemma associated with tattoo removal in sensitized patients. Additional therapeutic approaches are needed to provide patients with a safe means of tattoo removal while mitigating the risk of hypersensitivity reactions.

## Introduction

Allergic cutaneous reactions have been described with laser tattoo removal using Q-switch lasers, considered the gold standard in the field.^1,2^ But there is a paucity of literature describing the allergic cutaneous reactions to tattoo pigment following picosecond lasers, an emerging mainstay treatment modality in laser tattoo removal. Here, we report, to the best of our knowledge, the first case of an id reaction associated with allergic contact dermatitis (ACD) to red tattoo pigment following picosecond laser treatment.

## Case report

A 45-year-old, Asian, female was referred to our dermatology clinic for keloids following cosmetic picosecond laser tattoo removal.

The tattoo was placed several years without any reactions prior to the first laser session. The patient received all her tattoos from the same tattoo parlour. In May 2020, the patient tolerated the first session of laser tattoo removal on her left forearm. However, after the second session, in July 2020, she developed severe redness, swelling, and itchiness in the treated areas of her left forearm within days of the second laser session. She was reportedly treated for a superficial skin infection with antibiotics, and then for keloids with silicone sheets and topical corticosteroids. The use of topical corticosteroids prior to dermatology consultation may have altered the presentation of the rash to some degree. Rather than improving, she erupted into an itchy rash over her entire body. The patient was seen at our dermatology clinic in December 2020 after being diagnosed by other physicians with keloids between July and December 2020. She is known for hypothyroidism, for which she takes levothyroxine, but was otherwise healthy with no personal or family history of atopy.

**Figure 1.**
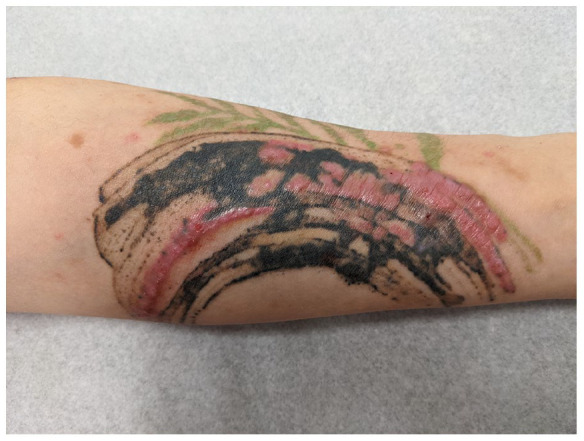
Cutaneous manifestation of localized allergic contact dermatitis to tattoo pigment following laser treatment. Patient’s laser-treated, left forearm (arch tattoo) shows erythematous, indurated plaque at site of laser tattoo removal.

**Figure 2.**
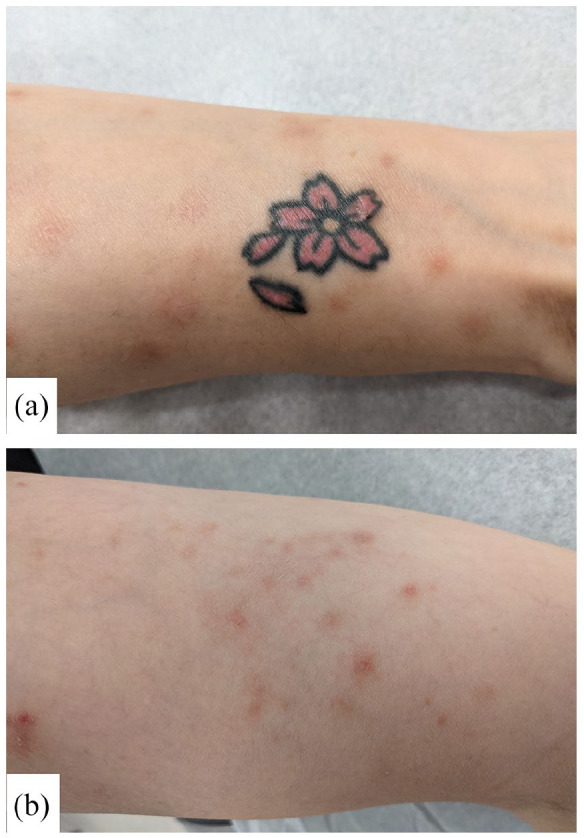
Cutaneous manifestation of id reaction following laser treatment. Patient’s untreated, right forearm (flower tattoo) showing erythematous, indurated papules (a) and proximal right forearm showing ill-defined, eczematous, erythematous papules, and plaques (b).

On exam, the left forearm (Figure 1) revealed a residual black and green tattoo with erythematous indurated plaques within the previously red areas. The right forearm (Figure 2(a)) revealed an untreated flower-shaped tattoo with similar, but less severe findings within the red petals. Otherwise, multiple eczematous, excoriated papules, and patches were scattered over her trunk and extremities (Figure 2(b)). There were no palpable abnormal lymph nodes.

Skin biopsies were obtained from the tattoos on both forearms. These biopsies revealed a dermal perivascular inflammatory infiltrate composed of lymphocytes and numerous eosinophils around exogenous red tattoo pigment. No granuloma was observed. Consequently, the patient was diagnosed with ACD to red tattoo pigment and id reaction.

Limited relief was achieved with clobetasol propionate ointment (0.05%) and intralesional triamcinolone acetonide (10 mg/mL). Although she responded to a trial of prednisone (20 mg/day tapered over 20 days), she relapsed shortly after it with disseminated lesions. Between May and June 2021, the patient underwent two treatments of serial fractionated ablative laser treatment. By late June 2021, the patient’s pruritus completely resolved, and the id reaction had resolved leaving post-inflammatory hyperpigmentation and pink patches.

## Discussion

ACD to red pigment in existing tattoos have been well-reported in the literature stemming from sensitivity to cinnabar (mercuric sulphide), historically, and even now with mercury-free organic pigments like azo dyes.^3–7^ In contrast, an id reaction following laser tattoo removal is not common. The current understanding of the pathophysiology of an id reaction centres around not only the spillover of superfluous cytokines generated in response to a local irritant or dermatitis eruption but also it involves micro- or nano-particles of exogenous material, like tattoo dye, that are spread via lymphatic or hematogenous means leading to distant skin autosensitization.^8^ In our case, we suspect that the initial laser removal treatment triggered the hypersensitivity reaction.

Treatments for an id reaction associated with ACD to tattoos include topical corticosteroids, topical calcineurin inhibitors, oral corticosteroids, intralesional corticosteroids, and procedural interventions like surgical removal and laser removal of the tattoo.^9^ Medical approaches are generally limited by the persistence of the sensitized tattoo pigment, acting as a contact allergen or hapten, driving the inflammatory id reaction. A surgical or laser approach would be curative in removing or breaking down the offending pigment as in the case of tattoos.^10^ Surgical approaches are limited by the size of the tattoo and the surgeon’s impression. As such, these treatments emphasize on a combination of inflammation management and tattoo pigment removal.

Common devices for laser tattoo removal include Q-switched lasers and the novel picosecond lasers. These lasers achieve tattoo removal by transepidermal elimination, tattoo pigment fracturing followed by removal via lymphatics and rephagocytosis of pigment by cells in the dermis. However, allergic cutaneous reactions to tattoo pigment following Q-switch laser removal have been reported.^1,2^ This was postulated to be a result of fragmented, extracellular tattoo pigment interacting with the immune system, and possibly even due to antigenic determinants being altered due to the photothermal effect of laser therapy, resulting in a hypersensitivity response.^2^

With picosecond lasers, there is a single report describing delayed anaphylaxis post-laser tattoo removal based on clinical history and physical examination.^11^ We add to the bare, literature associated with picosecond laser tattoo removal complications with our case of an id reaction associated with ACD to red tattoo pigment supported by clinicopathologic findings.

Given that the tattoo removing effect of picosecond lasers is achieved predominantly by a photoacoustic and mechanical mechanism rather than a photothermal reaction, our case adds additional evidence against the hypothesis that antigenic determinants in tattoo pigment are altered due to the photothermal effect of laser therapy and produce a hypersensitivity response. Our case adds further support for the pathophysiology of an id reaction in laser tattoo removal resulting from an immunologic response to extracellular particles of pigment. Also, in cases of laser tattoo removal performed with ablative and fractional CO_2_ laser, there have been reports of allergic cutaneous reactions where energy is not absorbed by the tattoo pigment to allow for the potential of antigenic alteration.^12–14^

Thus, tattoo removal in the setting of an id reaction associated with ACD to tattoo pigment is a challenging dilemma as there is a risk of triggering additional hypersensitivity reactions. There is a clear, unmet need for therapeutic approaches for tattoo removal in sensitized patients. There are reports of topical corticosteroids, oral corticosteroids, and oral antihistamines use post-laser as a mean to prevent hypersensitivity reactions in select cases.^1,2,12^ Therefore, prophylactic strategies are an unexplored area that would benefit from further research and may elucidate the answer to the dilemma of tattoo removal in sensitized patients.
